# Everyday Discrimination and Depressive Symptoms among Gujarati Adults: Gender Difference in the Role of Social Support

**DOI:** 10.3390/ijerph19148674

**Published:** 2022-07-16

**Authors:** Mieko Yoshihama, Jun Sung Hong, Yueqi Yan

**Affiliations:** 1 School of Social Work, University of Michigan, Ann Arbor, MI 48109, USA; miekoy@umich.edu; 2 School of Social Work, Wayne State University, Detroit, MI 48202, USA; 3 Health Science Research Institute, University of California Merced, Merced, CA 95343, USA; yyan105@ucmerced.edu

**Keywords:** racial discrimination, depressive symptoms, Asian Indian, gender difference, social support, sociocultural difference, Asian immigrants

## Abstract

Discrimination against Asians in the USA and its impact on their mental health are urgent public health concerns. Most research on discrimination against Asians has used aggregated Asian group samples. Focusing on Gujaratis, a specific subgroup of Asian Indians, the second-largest Asian group in the USA, this study examined the relationships between everyday discrimination and psychological distress and how they vary by gender. Data were collected via computer-assisted telephone interviews with a representative sample of 553 Gujaratis aged 18 to 65 years residing in a Midwestern state. Negative binomial regression analyses were conducted to examine how exposure to unfair treatment and three types of social support, respectively, was associated with depressive symptoms. For both women and men, unfair treatment was positively associated with depressive symptoms, controlling for sociodemographic characteristics. For women, but not for men, the incidence rate ratio became non-significant when adding social support measures to the model. All three social support measures for women, and only satisfaction with social support for men, were significantly associated with lower depressive symptoms. The findings highlight the need for further research on the role of different types of social support and gender differences, which can inform gender- and socioculturally-relevant intervention efforts.

## 1. Introduction

Violence against Asians has sharply increased and become more visible since the COVID-19 pandemic in 2020 [1,2,3,4,5], which makes research on discrimination and its impact on the psychological well-being of Asians in the USA timely and urgent. In response to this important public health problem, research on anti-Asian discrimination has increased in recent years, documenting a significant association between discrimination, especially unfair treatment, and psychological distress [6,7,8,9,10,11,12], consistent with research on African Americans and other minority groups [13,14,15,16,17,18,19,20,21,22]. However, there are several research gaps. First, many studies have relied on an aggregated group of Asians despite vast sociocultural variations. Second, studies of specific Asian groups tend to focus on relatively large groups such as Chinese [23,24,25,26], Filipinos [27,28,29,30], and Koreans [31,32,33,34]. Asian Indians, the second largest and the fastest-growing Asian group [35,36], have been under-researched, with the exception of Nadimpalli and colleagues’ [37] research on South Asians. Third, even among Asian Indians, sociocultural, linguistic, and religious differences are vast, which challenge the validity of studying Asian Indians as an aggregated group. Therefore, this study focuses on a specific ethnic group of Asian Indians: Gujaratis. Gujaratis are one of the largest Asian Indian populations in the USA; they trace their ancestry to Gujarat, India, and have their own language.

The effects of discrimination on mental health might vary by type and degree of available resources. Social support has been identified as one of such protective resources. Studies have shown that social support buffered the negative effects of discrimination on psychological well-being among various racial/ethnic minorities [38,39,40].

Social support has deep roots in psychological and sociological research [41,42,43,44,45]. While various models and theories have been put forward, the stress process model illuminates the mechanisms by which exposure to stressors leads to health outcomes in which social and personal resources, such as social support, operate to affect the stressor-health relationship [45,46,47,48,49]. Social support is understood to have either a direct positive effect on individuals’ psychological well-being or a buffering effect where social support alleviates the negative effects of stress in those under stress [41]. The existing research has often found that, compared with received social support, perceived availability of social support is more strongly associated with various measures of psychological distress [46,50]. 

Existing research on Asians has documented the buffering role of social support among those who have experienced discrimination [2,11,12]. A secondary analysis of the data from the National Latino and Asian American Study (NLAAS) found that perceived emotional support from family but not received or perceived support from friends moderated the relationship between unfair treatment and psychological distress [11]. Another secondary analysis of the data from NLAAS found that spousal support moderated the relationship between psychological distress and unfair treatment in general, but not racial discrimination specifically [12]. A recent study of a sample of Asian and Asian American adults recruited via an online platform found that social support significantly moderated the effect of discrimination on depressive symptoms [2]. Gee and colleagues’ [28] study of Filipinos in San Francisco and Honolulu found significant two-way interactions between city and instrumental support, emotional support, and unfair treatment, respectively, as well as a three-way interaction among instrumental support, unfair treatment, and city, suggesting strong effects of local context on the types of discriminatory behaviors encountered and resources available and/or used. Heeding the importance of local context, this study takes a place-based approach and examines the experience of unfair treatment, social support, and psychological distress among Gujaratis in a specific geographic context, an urban area in the Midwestern USA. 

Despite accumulating empirical data suggesting significant gender differences in various dimensions of interpersonal and social interactions [51,52,53], gender differences in the role of social support in the discrimination-distress relationship have not been well researched in studies of unfair treatment and mental health among Asians. Gender is typically included in the model as a control variable/covariate in examining the relationship between discrimination and mental health outcomes. This study examines whether and how the relationships between exposure to unfair treatment, social support, and mental health differ by gender. Focusing on a specific Asian Indian ethnic group, Gujaratis, in a specific locality, an urban area of a Midwestern state, we hypothesized that experiences of unfair treatment would be associated with overall depressive symptomatology. We also examined whether various types of social support would provide a buffer against depressive sequelae of unfair treatment and whether and how the association between social support and depressive symptoms varies by gender. 

## 2. Materials and Methods

### 2.1. Study Population and Context

Gujaratis trace their roots in the Indian state of Gujarat, which is located in the western region of India. They are one of the largest Asian Indian populations in the USA. According to an estimate, Gujarati, who make up 6% of the Indian population, constitute about 20% of the Asian Indian population in the USA [54]. While the largest concentration of Gujaratis and other Asian Indian groups are found in eastern states such as New York and New Jersey, midwestern states have seen a steady increase in the last decade [35]. Gujaratis are regarded as successful in the education and business spheres [55]. 

### 2.2. Sample and Procedure

Data were collected via computer-assisted telephone interviewing (CATI) from a survey research center of the first author’s institution. The inclusion criteria were as follows: being of Gujarati descent, being aged 18 to 65 years and residing in one of four urban counties of a midwestern state. Because the target population consisted of a small proportion of the general population, area sampling was not suitable for this group. Thus, we drew a random sample using an ethnic surname list compiled by a sampling company, which had been tested for use in research with various racial and ethnic minority groups, including South Asians [56,57].

An introductory letter was mailed to the randomly selected households. An interviewer from the university’s survey center called each household to identify an eligible participant. If multiple eligible individuals resided in a household, a CATI- generated random number procedure was used to select one individual. Consent was obtained at the beginning of the interview. The interview schedule was translated to Gujaratis using a back-translation method, and both the English and Gujarati language interview schedules were available through a CATI system. A total of 553 individuals participated in the interview; the response rate was 65.8%. The interviews lasted for 76.9 min on average, and the participants received USD 25 for their participation. The study was approved by the Institutional Review Board of the first author’s affiliation, and a Certificate of Confidentiality was obtained from the National Institutes of Health. 

### 2.3. Measures

#### 2.3.1. Unfair Treatment

We assessed exposure to eight types of unfair treatment during the past six months using the Everyday Discrimination Scale [18], a widely used measure (sample items: “received poorer service at restaurants or stores,” “called names or insulted,” and “were threatened or harassed”) with the following modifications. First, we combined two items (being treated with less courtesy and being treated with less respect) into one item (being treated with less courtesy or respect), a modification consistent with the Everyday Discrimination Scale (Short Version) [58]. We used a 5-point scale (0 = never, 1 = rarely, 2 = sometimes, 3 = often, 4 = always) by modifying the 5-point scale used in the Detroit Area Study (1 = never, 2 = hardly ever, 3 = not too often, 4 = fairly often, 5 = very often) [59] to simplify the wording and avoid using “fairly often” and “very often,” which can be difficult to distinguish over telephone interviews. We used a timeframe of the last six months to capture recent exposure to unfair treatment rather than a lifetime. The Everyday Discrimination Scale has been used with various Asian groups, including Asian Indians in the NLAAS, as part of the Collaborative Psychiatric Epidemiology Surveys [60], which included a sample of various Asian groups, including Asian Indians. We summed the responses for each item to create a total score of unfair treatment, with greater values indicating higher levels of unfair treatment. The Cronbach’s Alpha for these eight items was 0.805.

#### 2.3.2. Depressive Symptoms

Past-week experience of depressive symptoms was measured by the Center for Epidemiological Studies Depression Scale (CES-D) [61], a widely used measure of depressive symptomatology. Its 20 items assess the frequency of symptoms experienced during the previous week (sample items: “I was bothered by things that don’t usually bother me,” “I did not feel like eating: my appetite was poor,” and “I felt I could not shake off the blues”). Response options ranged from 0 = rarely or none of the time to 3 = 5 to 7 days. The responses for each item were summed. Higher total scores indicated more depressive symptomatology. This scale was previously used with South Asian populations in the USA [60]; while Rahman and Rollock [62] did not report psychometrics, studies in various Asian countries [63,64] reported good validity and reliability of the CES-D and found factor structures similar to those found in general populations in the USA [65]. The Cronbach’s Alpha for this sample was 0.794.

#### 2.3.3. Social Support

Three types of social support were measured: frequency of contact, availability, and satisfaction. The frequency of contact with family members (those not currently living together) and friends was measured by two items adapted from NLAAS [60] using a 7-point scale ranging from 1 = never to 7 = nearly every day. Responses to the two items were then averaged. Estimating the reliability coefficient for two items is challenging. In general, Cronbach’s Alphas underestimate reliability, sometimes dramatically, and Spearman-Brown coefficients are generally considered the most appropriate reliability statistic for a two-item scale [66,67]. The SpearmanBrown coefficient for these two items was 0.493, indicating that these two items are moderately correlated. 

Available social support was measured using four questions adapted from the MOS-SSS4 [68]. The four items tapped into four domains corresponding to the four factors found in the original Medical Outcomes Study Social Support Survey (MOS-SSS) [69]: emotional-informational support, tangible support, affectionate support, and positive social interaction. Using a 5-point scale (ranging from 1 = none of the time to 5 = all the time), respondents reported the perceived availability of each type of support: someone to turn to for suggestions about how to deal with personal problems (emotional-informational support); someone to help with daily chores if you were sick (tangible support); someone to do something enjoyable with (positive social interaction); someone who shows love and affection (affectionate support). The last item was adopted from the original MOS-SSS instead of the MOS-SSS4 because the MOS-SSS4 item—someone to love and make you feel wanted—did not perform well in a pilot test. The responses to the four items were averaged. The Cronbach’s Alpha for the four items of available social support was 0.784.

The third measure of social support pertains to the degree of satisfaction with available social support, which is assessed in the Social Support Questionnaire [70,71]. Because we assessed the frequency of contact with family members and friends (the first measure of social support), we assessed the degree of satisfaction with available social support from family and friends, respectively. Responses on a 4-point scale (ranging from 1 = not satisfied at all to 4 = very satisfied) for each item were averaged. The Spearman-Brown coefficient for the two items was 0.668.

#### 2.3.4. Demographics

As covariates we included age groups (1 = 18 to 29, 2 = 30 to 39, 3 = 40 to 49, 4 = 50 to 59, 5 = 60+); the highest educational degree obtained (1 = less than bachelor’s degree, 2 = bachelor’s degree, 3 = graduate degree); employment status (0 = not employed, 1 = working part time, 2 = working full time); marital status (0 = not married, 1 = married), financial difficulty (0 = no, 1 = yes); and length of residency in the USA. The last measure used the % of years lived in the USA, which was calculated based on the respondent’s age at interview, country of birth, and age at immigration; we used the percentage, instead of the number, of years spent in the USA to avoid confounding with the participant’s chronological age.

### 2.4. Analytical Approach 

The primary purpose of this study was to examine the relationship between experiences of unfair treatment, social support, and depressive symptoms. The dependent variable (CES-D scores) could take only non-negative integer values, the variance was considerably larger than the mean, and the distribution was highly skewed with many small values and a few large values (overdispersed). Therefore, we used negative binomial regression [72]. Analyses were conducted using SPSS Statistics for Windows, Version 28.0.1.0 [73].

We conducted skewness and kurtosis tests to assess the assumption of normal distribution of CES-D scores. The CES-D score violated the assumption (*p* < 0.001). The integer score was overdispersed, and the mean was much lower than its variance, suggesting a negative binomial model would be appropriate. In the negative binomial regression (NBR) model, the log-transformed over-dispersion parameter for each independent variable or covariate was estimated. Incidence rate ratios (IRRs) with 95% confidence intervals were reported. An IRR greater than 1 indicated that, for example, a 1-unit increase in unfair treatment was associated with a higher depression score. 

We conducted separate analyses for women and men. First, we performed bivariate NBR analyses to examine the association of the discrimination score or each social support measure with the depression score among women. Next, separate partially adjusted NBR analyses were used to examine whether the association of each independent variable of interest with depression scores remained significant while controlling for demographic characteristics. Finally, we included all independent variables and demographic variables to run a multivariate (or fully adjusted) NBR model to generate a fully adjusted estimate of the effect of unfair treatment experiences and social support measures on depression scores, controlling for demographic characteristics. In addition, moderation terms of unfair treatment with social support measures were added to the fully adjusted model to examine whether any social support measures buffer the adverse effects of unfair treatment on depression scores among women while controlling for demographic variables. The same set of analyses was subsequently performed for men. 

Multicollinearity among three social support measures was assessed using tolerance tests after each NBR model; a variance inflation factor (VIF) value greater than 10 suggests a strong relationship among the predictors and merits further investigation. None of the VIFs for the three social support measures was higher than 10. The highest correlation was found between the availability of social support and satisfaction with available social support (*r* = 0.457, Tolerance = 0.7911, VIF = 1.264), indicating an absence of multicollinearity concerns. Using the conditional power calculation method, a sample size of 273 for women and 280 for men achieved 97% and 98% power, respectively, to detect an R^2^ of 0.06 (an attributed small effect of R^2^ for each independent variable [74] attributed to 3 independent variable(s) using an F-Test with a significance level (alpha) of 0.05. The variables tested were adjusted for an additional 6 covariate(s) which had a combined R^2^ of 0.12 (an attributed small effect of R^2^ for each covariate [74] by themselves. 

## 3. Results

### 3.1. Respondent Characteristics

As shown in Table 1, on average, respondents were 45 years old (women, M = 44.63, SD = 12.34; men, M = 45.00, SD = 12.03) and had lived in the USA for half of their lives (women, M = 48.6%; men, M = 50.3%). The majority of respondents, both women and men, had a bachelor’s degree or higher, with men more likely to hold a graduate degree (30.4% of women vs. 57.1% of men, *p* < 0.001). Most respondents were employed at the time of the interview, with 46% of women and 75% of men working full time (*p* < 0.001). Approximately one-third of respondents reported some degree of financial difficulties (35.4% of women vs. 30.7% of men, *p* = 0.24). The majority of respondents of both genders were married at the time of the interview (87.2% of women vs. 85.7% of men, *p* = 0.62); those who were not married at the time of the interview reported their relationship status as “never married” (*n* = 56), divorced or separated (*n* = 13), and widowed (*n* = 6). In the absence of demographic profiles of Gujaratis in the study area (or the state or national level), the degree of representativeness cannot be numerically assessed. However, the high educational attainment, as well as greater labor participation among men reported in this study, is consistent with data available for Asian Indians as a group [75].

### 3.2. Reports of Unfair Treatment, Social Support, and Depressive Symptoms

About 65% to 75% of the respondents (64.1% of women and 74.6% of men, *p* = 0.007) reported having experienced one or more types of unfair treatment. Women reported a lower frequency of unfair treatment compared with men. Among those who reported unfair treatment, the mean frequency of unfair treatment experienced was 2.76 (SD = 3.23) for women and 3.73 (SD = 3.71) for men, which was significantly different *(p* = 0.003). 

The mean CES-D scores were higher for women (M = 5.84, SD = 6.64) than men (M = 4.75, SD = 5.18, *p* = 0.03). For both women and men, their responses to the three measures of social support showed that, in general, they had frequent contact with family and friends (M = 5.74–5.81, between a few times a month and at least once a week); the average availability of support (i.e., emotional-informational, tangible, positive social interaction, and affectionate) was 4.13 and 4.03 for women and men, respectively; and the average level of satisfaction with social supports among individuals of both genders was between somewhat and very satisfied with the available social support (3.65 for women and 3.64 for men). No significant gender differences were found in all three types of social support reported. 

### 3.3. Relationship between Unfair Treatment and Depressive Symptoms

As shown in Table 2, unfair treatment was significantly associated with higher depression scores in women in both bivariate (IRR = 1.08, *p* < 0.001) and partially adjusted models (IRR = 1.06, *p* = 0.003). However, the incidence rate ratio became non-significant when we added social support measures (*p* = 0.17). For women, all three social support measures were significantly associated with lower depression scores in the bivariate, partially adjusted, and fully adjusted models, with satisfaction with social support being the strongest predictor of lower depression scores (IRR = 0.44, *p* < 0.001 for bivariate; IRR = 0.52, *p* < 0.001 for partially adjusted; IRR = 0.67, *p* = 0.009 for fully adjusted). 

For men, unlike women, the significant association between unfair treatment and depressive symptoms remained even when all three measures of social support were included in the model (IRR = 1.07, *p* < 0.001 for bivariate; IRR = 1.07, *p* < 0.001 for partially adjusted; IRR = 1.05, *p* = 0.012 for fully adjusted). As for social support, availability of social support was significantly associated with lower depressive symptoms in the bivariate model (IRR = 0.78, *p* = 0.004) and the partially adjusted model (IRR = 0.81, *p* = 0.02) but not in the fully adjusted model (IRR = 0.96, *p* = 0.64). The frequency of contact with family/friends did not act as a protective factor in reducing depression scores in any of the models. In contrast, satisfaction with social support was negatively associated with depressive symptoms in all three models (IRR = 0.60, *p* < 0.001 for bivariate; IRR = 0.61, *p* < 0.001 for partially adjusted; IRR = 0.67, *p* = 0.004 for fully adjusted). 

We also examined whether social support moderates the relationship between unfair treatment and depressive symptoms by entering the interaction between unfair treatment and each type of social support. None of the interaction terms were significant for both women and men.

## 4. Discussion

Little is known about discrimination and its mental health effects in the lives of Gujarati individuals in the USA. To date, this study is the first empirical investigation of the association between unfair treatment and depressive symptoms in this previously under-studied population. A majority of both women and men reported experiencing unfair treatment. This is comparable with Chae and colleagues’ [76] study of Asians using the NLAAS data, which found that the majority of the sample (74.2%) reported receiving unfair treatment. These findings collectively point to a fairly common occurrence of unfair treatment in the lives of Gujaratis and other Asians in the USA, a nation afflicted with racial/ethnic discrimination and xenophobia.

As hypothesized, Gujarati women and men who had experienced unfair treatment during the previous six months reported significantly higher past-week depressive symptoms. The significant relationship corroborates studies of an aggregated group of Asians [11,12] and specific Asian ethnic groups [23]. The finding is also consistent with studies of other racial/ethnic groups [19], suggesting an effect of unfair treatment that cuts across racial/ethnic boundaries. 

This study’s findings call for increased attention to prevalent exposure to unfair treatment and its psychological sequelae among Asians in practice. In clinical settings, to assess depressive symptoms and provide timely care, healthcare practitioners and allied professionals should inquire about exposure to unfair treatment as a possible contributing factor to depressive symptoms. The increased incidents of harassment and violence against Asians during the COVID-19 pandemic [2,3] make this type of practice even more critical. In light of existing research pointing to low help-seeking from mental health services among Asians [77,78], including Asian Indians (though not specific to Gujaratis) [79], in addition to mental health services, primary care and alternative medicines that are frequently used by Gujaratis may be suitable points of intervention. 

For women, social support, whether related to contact frequency, perceived availability, or satisfaction, was associated with lower depressive symptoms. For men, it was only satisfaction with social support that was significantly related to depressive symptoms. The lack of significant interaction between unfair treatment and any of the three types of social support examined is somewhat surprising. Previous studies of an aggregated group of Asians have reported buffering effects of social support among individuals exposed to discrimination [11,12]. While further examination is needed, it is possible that social support, especially satisfaction with available social support, has an “omnibus” and a direct impact on individuals’ psychological well-being regardless of exposure to unfair treatment; or exposure to unfair treatment is so distressing that social support does not make much of a difference; or both. Of the three measures of social support, satisfaction with available social support was more strongly associated with lower depressive symptoms for both women and men. This suggests that attempting to increase the frequency of contact or availability of support alone will not be effective in decreasing depressive symptoms. Because what might increase individuals’ sense of satisfaction with available support likely varies by their social locations and other contextual factors (e.g., gender, age, marital/relationship status, immigration status), in order to develop an effective intervention, more research is necessary. Toward this end, qualitative analyses may be helpful. 

Observed gender differences in the association between social support and depressive symptoms deserve attention. While no studies of unfair treatment and depressive symptoms in Asians documented gender differences in the effect of social support, a study of Asian college students [80] found significant gender differences in how coping behaviors mediated the relationship between racism and racism-related stress. A secondary analysis of the NLAAS data [81] found a gender difference in the threshold level at which discrimination was associated with negative physical and mental health, with women having a lower threshold. The authors concluded that “failing to examine women and men separately in discrimination research may no longer be appropriate among the Asian-American population” (p. 350). The current study’s findings of significant gender differences in the association between social support and depressive symptoms provide additional empirical data with which to advocate for gender- and socioculturally appropriate treatment plans for individuals experiencing perceived discrimination and adverse mental health outcomes. More research is needed to develop effective prevention and intervention programs responsive to differences that might exist on the basis of gender. 

A major strength of our study is the use of a community-based random sample comprising a single ethnic group. Rather than an aggregated group of Asians, analysis of a disaggregated ethnic group is consistent with recommendations by various scholars [82,83]. Furthermore, instead of controlling for gender, separate analyses by gender helped elucidate important gender differences. This study also focused on a specific locality. While a study of larger scope (e.g., a national study) may provide more generalizable data, the importance of attending to local contexts was demonstrated by Gee and colleagues’ [28] study of Filipinos in San Francisco and Honolulu; they state, “Local contexts may influence the types of treatment encountered by members of ethnic minority groups, as well as their resources. These factors, in turn, may have implications for health disparities and well-being” (p. 677). As such, the extent to which this study’s findings are applicable to Gujaratis in other locations (e.g., states of New York and New Jersey) remains unknown, requiring location-specific investigation. Lastly, assessing three types of social support extends the previous research, and although social support has long been identified as a salient protective factor for racial minorities and immigrants, consideration of various dimensions of social support can facilitate identifying relevant and necessary resources for individuals experiencing discrimination and associated mental health problems. 

Conducting research with an under-researched or under-represented population group is challenging because using the existing scales with known reliability and validity based on mainstream population groups does not always warrant the validity of results. We have made necessary modifications and adaptations to the existing instruments as done by many researchers to suit the study populations and specific study aims and context. These modifications were informed by careful and critical reviews of existing research, consultations with experts, including those who have been involved in and familiar with the Detroit Area Study and minority populations, ongoing discussions with community informants, and a series of pilot tests and pretests. While our modifications were minor and the measures met the criteria of sound reliability and face validity, future studies are needed to assess the validity of the refined version that targets diverse populations. 

The limitations of this study include the use of a cross-sectional design and respondents’ retrospective self-report. Despite the cross-sectional nature, the current study assessed the past-week symptomatology and exposure to unfair treatment during the previous six months. These two timeframes provide some limited support for the contention that exposure to unfair treatment likely happened before or simultaneously with depressive symptom development. Nonetheless, a study of a longitudinal design is needed to examine changes in depressive symptomatology over time relative to exposure to unfair treatment. Not only would such studies be costly but also methodologically and ethically challenging. Methodologically, the prevalent and recurrent nature of both the exposure to unfair treatment and the experience of depressive symptoms makes it difficult to tease out whether exposure to unfair treatment preceded symptom development. Because of the significant link between unfair treatment and depressive symptoms, which, especially if untreated, can have negative sequelae, including suicidal and self-injurious behaviors [84], following individuals over time for data collection without providing timely intervention poses ethical issues. Depression is a leading cause of disability worldwide [85] and is a consistent and strong correlate of suicidal behaviors [84]. Though designing methodologically and ethically sound research is challenging, the prevalent nature of unfair treatment and its association with depressive symptoms call for further research. 

## 5. Conclusions

Experiencing unfair treatment is associated with greater levels of depressive symptoms for Gujarati women and men. The role of social support differs by gender. Prevention and treatment programs that are responsive to unique sociocultural characteristics and gender are urgently needed. The societal costs of undetected and untreated depression are too high. Now is the time to expand research on depression and everyday discrimination, such as unfair treatment among under-studied population groups, which could contribute to better psychological well-being.

## Figures and Tables

**Table 1:** Respondent Characteristics by Gender (*N* = 553).

	Women (*n* = 273)		Men (*n* = 280)		Statistical Test
	*n*	*%*	*n*	*%*	
Age, mean (*SD*)	44.63 (12.34)		45 (12.03)		
18–29 years	34	12.50%	28	10.00%	ꭓ^2^ (4, 552) = 1.240, *p* = 0.87
30–39 years	68	24.90%	69	24.70%	
40–49 years	69	25.30%	73	26.20%	
50–59	65	23.80%	65	23.30%	
60+	37	13.60%	44	15.80%	
Educational levels					ꭓ^2^ (2, 553) = 40.133, *p* < 0.001
<Bachelor’s degree	58	21.20%	36	12.90%	
Bachelor’s degree	132	48.40%	84	30.00%	
Graduate degree	83	30.40%	160	57.10%	
Employment status					ꭓ^2^ (2, 553) = 49.530, *p* < 0.001
Not working	86	22.30%	40	14.30%	
Working part-time	61	22.30%	29	10.40%	
Working full-time	126	46.20%	211	75.40%	
Financial difficulty					ꭓ^2^ (1, 548) = 1.390, *p* = 0.24
No difficulty	175	64.60%	192	69.30%	
Some level of difficulty	96	35.40%	85	30.70%	
Marital status					ꭓ^2^ (1, 553) = 0.253, *p* = 0.62
Not married	35	12.80%	40	14.30%	
Married	238	87.20%	240	85.70%	
% of years lived in the USA, M (*SD*)	48.61	(25.20)	50.33	(24.52)	t (545.475) = −0.81, *p* = 0.18
Reported at least one type of unfair treatment	175	64.10%	209	74.60%	ꭓ^2^ (1, 553) = 7.24, *p* = 0.007
	*M*	*SD*	M	*SD*	
Unfair treatment	2.76	3.23	3.73	3.71	t (420512.49) = 2.14, *p* = 0.003
CES-D scores	5.84	6.64	4.75	5.18	t (544.18) = −3.26, *p* = 0.001
Frequency of contact	5.74	0.93	5.81	0.84	t (541.99) = −0.94, *p* = 0.19
Availability of social support	4.13	0.74	4.03	0.79	t (548.91) = 1.63, *p* = 0.26
Satisfaction with social support	3.65	0.44	3.64	0.54	t (532.23) = 0.117, *p* = 0.06

**Table 2:** Negative Binomial Regression Predicting CES-D Scores.

	Bivariate Model		Partially Adjusted Model *		Fully Adjusted Model *	
	IRR (95% CI)	*p* Value	IRR (95% CI)	*p* Value	IRR (95% CI)	*p* Value
**Women**						
Unfair treatment	1.08 (1.038, 1.128)	<0.001	1.06 (1.020, 1.104)	0.003	1.03 (0.988, 1.069)	0.17
Frequency of contact	0.78 (0.670, 0.907)	0.001	0.79 (0.690, 0.908)	0.001	0.85 (0.745, 0.957)	0.008
Availability of social support	0.65 (0.537, 0.779)	<0.001	0.67 (0.563, 0.804)	0.001	0.79 (0.654, 0.942)	0.009
Satisfaction with social support	0.44 (0.328, 0.582)	<0.001	0.52 (0.387, 0.684)	0.001	0.67 (0.494, 0.904)	0.009
**Men**						
Unfair treatment	1.07 (1.030, 1.104)	<0.001	1.07 (1.028, 1.107)	<0.001	1.05 (1.011, 1.090)	0.012
Frequency of contact	0.94 (0.801, 1.094)	0.41	0.97 (0.819, 1.137)	0.67	1.02 (0.871, 1.192)	0.82
Availability of social support	0.78 (0.660, 0.927)	0.004	0.81 (0.672, 0.966)	0.02	0.96 (0.788, 1.159)	0.64
Satisfaction with social support	0.60 (0.465, 0.771)	<0.001	0.61 (0.470, 0.791)	0.001	0.67 (0.509, 0.880)	0.004

* Age groups, education level, employment status, financial difficulty, marital status, and % of years lived in the USA were controlled in the model.

## Data Availability

The data presented in this study are available upon request from the corresponding author. The data are not publicly available due to privacy and confidentiality reasons.
